# A strategy for residual error modeling incorporating scedasticity of variance and distribution shape

**DOI:** 10.1007/s10928-015-9460-y

**Published:** 2015-12-17

**Authors:** Anne-Gaëlle Dosne, Martin Bergstrand, Mats O. Karlsson

**Affiliations:** Department of Pharmaceutical Biosciences, Uppsala University, P.O. Box 591, 751 24 Uppsala, Sweden

**Keywords:** Residual error, Transform-both-sides, Skewness, Heteroscedasticity, Heavy tails, t-Distribution

## Abstract

Nonlinear mixed effects models parameters are commonly estimated using maximum likelihood. The properties of these estimators depend on the assumption that residual errors are independent and normally distributed with mean zero and correctly defined variance. Violations of this assumption can cause bias in parameter estimates, invalidate the likelihood ratio test and preclude simulation of real-life like data. The choice of error model is mostly done on a case-by-case basis from a limited set of commonly used models. In this work, two strategies are proposed to extend and unify residual error modeling: a dynamic transform-both-sides approach combined with a power error model (dTBS) capable of handling skewed and/or heteroscedastic residuals, and a t-distributed residual error model allowing for symmetric heavy tails. Ten published pharmacokinetic and pharmacodynamic models as well as stochastic simulation and estimation were used to evaluate the two approaches. dTBS always led to significant improvements in objective function value, with most examples displaying some degree of right-skewness and variances proportional to predictions raised to powers between 0 and 1. The t-distribution led to significant improvement for 5 out of 10 models with degrees of freedom between 3 and 9. Six models were most improved by the t-distribution while four models benefited more from dTBS. Changes in other model parameter estimates were observed. In conclusion, the use of dTBS and/or t-distribution models provides a flexible and easy-to-use framework capable of characterizing all commonly encountered residual error distributions.

**Electronic supplementary material:**

The online version of this article (doi:10.1007/s10928-015-9460-y) contains supplementary material, which is available to authorized users.

## Introduction

Population modeling is increasingly used to analyze data arising from clinical trials. Nonlinear mixed effects models (NLMEM) enable simultaneous analysis of data gathered from all study patients with the aim of determining an underlying structural model driving the observations as well as characterizing inter-individual variability (IIV), which explains why different individuals can show different responses to a given drug. In most cases, some information is available beforehand about the structural model based on the knowledge on the underlying system. For example, models of different systems which have been developed in multiple disease areas [1, 2] can be applied and adapted to new drug entities. Information about IIV can also be available in terms of physiological features of the study population such as polymorphism in metabolic enzymes or age-related alterations of particular organs. Another layer of variability, typically referred to as residual unexplained variability (RUV), accounts for all remaining variability which is not explained by the structural or parameter-variability model parts. This RUV arises for example from physiological intra-individual variation, assay error, errors in independent variables and model misspecification. The residual error thus aggregates multiple processes which causes and consequences are poorly defined. Despite this complexity, in the modeling process RUV is assumed to be normally distributed with mean zero and a defined variance, which can be homoscedastic, i.e., constant over model predictions, or heteroscedastic, i.e., dependent on model predictions.

Misspecifications of the residual error model impact both the estimation of and the simulation from NLMEM. Let us considerer first the impact of a misspecification of the scedasticity. Maximum likelihood (ML) estimation utilizes the expected variance of the modeled outcome in order to compute the likelihood to maximize. Estimation of model parameters will thus depend on the residual variance model, and parameter estimates may be biased if the wrong variance model is chosen [3]. Inference using the computed likelihood or standard errors (SEs) of parameter estimates resulting from such a fit may also be invalidated [4, 5]. Despite these risks, testing for heteroscedasticity in NLMEM is often limited to a plot of residuals or alternatively their absolute or squared values versus model predictions. More advanced tests including the family of score tests [6] have been proposed in fields such as econometrics but have not penetrated the field of pharmacometrics. When simulating from a NLMEM, predictions will clearly depend on the defined relationship between residuals and predictions, which is particularly important when simulating data outside of the range of the data used for estimation. The impact of a misspecification of the distribution shape of the residual error is less straightforward. If the scedasticity is correctly specified but the normality assumption is not verified, estimation comes back to extended least squares [7]. Resulting estimators in mixed effects models may be biased and their uncertainty may not be appropriate [8]. Simulations using a NLMEM ignoring skewness will often underestimate variability by simulating less extreme values.

It is thus important to address potential misspecifications of the residual error model. Even though elaborate models have been developed and advocated [3], residual error modeling is still mostly done on a case-by-case basis using a limited set of models. Scedasticity could easily be extended from the commonly used additive, proportional or combined error models to power models, which include and expand on the former. Skewness could be addressed based on an extension of the transform-both-sides (TBS) approach, in which both the observations and the predictions are transformed so that the resulting residuals on the transformed scale are normally distributed. An advantage of the TBS approach is that model parameters are still estimated on the same scale as when using untransformed data. The most common TBS approach is the log-transformation, which is used when observations span multiple orders of magnitude and/or are bound to be positive, such as for drug concentrations in pharmacokinetic (PK) studies [9], or when the endpoint relates to a ratio scale, such as percentage change from baseline for pharmacodynamic (PD) studies [10]. The log-transformation is actually a specific case of the Box–Cox power family of transformations [11], which have been used to describe chemotherapy-induced myelosuppression in cancer patients [12] for example. Because a major factor limiting the use of transformations so far has been that it was impossible to quantitatively select the best transformation due to the dependence of the likelihood to the value of the transformed data, approaches enabling the estimation of the transformation parameter directly would be an important asset in RUV modeling. Lastly, the use of distributions other than the normal distribution has been proposed [13] but remains underused, notably due to their unavailability in standard PK–PD software.

We propose that two approaches shall be considered in order to extend supported scedasticity relationships and enable objective selection of the distribution shape. The first approach, referred to as the dynamic TBS (dTBS), is based on the classical TBS approach but allows both the shape and the scedasticity parameters to be estimated. The second approach replaces the assumption of normality of the residuals by the assumption of a different parametric distribution. In this paper we investigated the assumption that the residuals arise from a Student’s t-distribution with estimated degree of freedom, which allows for heavier tails than the normal distribution if needed. The aim of the present work was to investigate the dTBS and t-distribution approaches using both real data examples and simulations in order to provide a comprehensive framework for characterization of the residual error model in NLMEM.

## Methods

### General framework

Let us first describe a general framework for the modeling of PK–PD data. A generic model for a given set of observed data *Y*, which depends on model parameters *θ* and independent variables *x*, is defined in Eq. 1. Indexes relative to individuals, time and/or other independent variables were omitted for simplification purposes. The variance of *Y* according to this model is defined in Eq. 2. Most commonly used residual error models are variants of the linear (i.e., *ζ* = 1) slope–intercept model, namely the additive error model ($\sigma_{slope}^{2} = 0$), the proportional error model ($\sigma_{intercept}^{2} = 0$) and the combined error model ($\sigma_{slope}^{2} \ne 0$ and $\sigma_{intercept}^{2} \ne 0$). However, the power parameter *ζ* can also be estimated [14] to allow for nonlinear heteroscedastic residual variances.

1 $$Y = f(x,\;\theta ) + f(x,\;\theta )^{\zeta } \times \varepsilon_{slope } + \varepsilon_{intercept} $$

2 $$Var(Y) = f(x,\;\theta )^{2\zeta } \times \sigma_{slope}^{2} + \sigma_{intercept}^{2} $$

where *Y* is observed data with variance *Var*(*Y*) given the model, *f* is a function describing the structural model, *x* are independent variables, *θ* are model parameters, *ζ* is a power parameter and *ε*_*slope*_ and *ε*_*intercept*_ are assumed independent with mean 0 and variance $\sigma_{slope}^{2}$ and $\sigma_{intercept}^{2} ,$ respectively.

The set of parameters which fit a set of data best are estimated by minimizing minus two times the log-likelihood, also referred to as objective function value (OFV), which is computed assuming a normal distribution of the residual error terms (Eq. 3). Minimization of the OFV leads to ML estimates only if the scedasticity and the distribution shape of the residual error are correctly specified, i.e., if *Var*(*Y*) is appropriate and the residuals are normally distributed.

3 $$OFV = {-} 2LL_{Y} = \log (Var(Y)) + \frac{{(Y - f(\theta ,\;x))^{2} }}{Var(Y)}$$

where −2*LL*_*Y*_ is minus two times the log-likelihood of the observed data *Y*, *Var*(*Y*) is the variance of *Y* given the model and *f*(*θ*, *x*) is the expectation of *Y* given the model.

### dTBS

If the distribution of the residuals appears skewed on the untransformed scale, ML estimation can still be performed using the TBS approach. Observations and predictions are transformed so that residuals, obtained as the differences between the transformed observations and the transformed predictions, can be assumed normally distributed. The Box–Cox transformation (Eq. 4) is a commonly used transformation to reach this goal, with its shape parameter *λ* accounting for skewness. A value of *λ* greater than 1 indicates left skewness, while a value of *λ* lower than 1 indicates right skewness. Special cases are a value of 1, which indicates no skewness, i.e., normally distributed residuals on the untransformed scale, and a value of 0, which corresponds to a log-normal distribution. The Box–Cox transformation can also handle negative observations by adding a constant to all observations before transformations to ensure their positivity. Whereas the traditional TBS approach assumes that the transformed variable has a constant or homoscedastic variance, we propose to combine the TBS approach with a more flexible power residual variance model. The resulting dTBS model and its corresponding variance are defined in Eqs. 5 and 6. The power was chosen to apply to the untransformed prediction. As will be shown in Eq. 7, the Box–Cox transformation does not only adjust for skewness but also implies a fixed power relationship to the untransformed prediction. The estimated power thus needed to be applied to the untransformed prediction if one wanted to adjust separately for shape and scedasticity.

4 $$\begin{array}{cc} {h(X,\;\lambda ) = \ln (X) } & {{\text{if}}\;\lambda = 0} \\ {h(X,\;\lambda ) = \frac{{X^{\lambda } - 1}}{\lambda }} & {\text{otherwise}} \\ \end{array}$$

5 $$h(Y,\;\lambda ) = h(f(x,\;\theta ),\;\lambda ) + f(x,\;\theta )^{\zeta } \times \varepsilon $$

6 $$Var(h(Y,\;\lambda )) = f(x,\;\theta )^{2\zeta } \times \sigma^{2} $$

where *h* is the Box–Cox transformation function, *X* is a random variable, *λ* is the shape parameter of the Box–Cox transformation, *ζ* is a power parameter and *ε* are assumed independent with mean 0 and variance *σ*^2^.

Structural model parameters estimated using the dTBS model will have exactly the same interpretation as if no transformation had been used, which is a major advantage of this approach. However, parameters related to the residual error model do not translate directly to the original untransformed scale. According to Taylor series expansion, the variance of the untransformed data *Y* can be approximated from the variance of the transformed data *h*(*Y*, *λ*) as stated in Eq. 7 and is approximately proportional to the 1 − λ + ζ power of the model predictions. This is widely known for log-transformed data, where an additive error on the transformed scale (λ = 0 and ζ = 0) is approximated by a proportional error model on the untransformed scale. From Eq. 7, it is apparent that the shape parameter λ does not only correct for skewness, but also influences scedasticity on the untransformed scale. It is then easily understood than if ζ is fixed, λ will need to adjust both the scedasticity and the skewness. However it is not guaranteed that a single value of λ can lead to both adequate scedasticity and normally distributed residuals. The addition of a power parameter is thus truly necessary to be able to address both aspects. Note that the dTBS model may be reparameterized using ζ = λ + δ, with δ estimated instead of ζ in order to decrease the correlation between estimated dTBS parameters. The dTBS model thus comprises the additive (λ = 1, ζ = −1), proportional (λ = 1, ζ = 1) and additive on log (λ = 0, ζ = 0) error models.

7 $$Var(Y) \approx Var(h(Y,\;\lambda )) \times \frac{dh(f(\theta ,\;x),\;\lambda )}{df(\theta ,\;x)} \approx f(\theta ,\;x)^{2\zeta } \times \sigma^{2} \times f(\theta ,\;x)^{2(1 - \lambda )} \approx \sigma^{2} \times f(\theta ,\;x)^{2(1 - \lambda + \zeta )} $$

Dynamic estimation of the new error model parameters λ and ζ needed to be addressed. Whereas no modification of the ML algorithm as implemented in NONMEM [15] is required to estimate the power parameter ζ, a modification of the procedure is mandatory to estimate the shape parameter λ of the Box–Cox transformation. Indeed, the calculated log-likelihood corresponds to the log-likelihood of the transformed data (Eq. 8). This quantity changes scale depending on the value of λ and thus cannot be used for parameter estimation when λ itself is estimated.

8 $${-} 2LL_{h(Y,\;\lambda )} = \log (Var(h(Y,\;\lambda ))) + \frac{{(h(Y,\;\lambda ) - h(f(\theta ,\;x),\;\lambda ))^{2} }}{Var(h(Y,\;\lambda ))}$$

Modifying the minimization criterion to reflect the likelihood of the data on the untransformed scale instead of the transformed scale enables quantitative comparison between transformations and thus dynamic estimation of λ [16–18]. The likelihood of the untransformed data can be calculated from the likelihood of the transformed data according to the change of variable formula (Eq. 9). The criteria to use for ML estimation can then be derived (Eq. 10).

9 $$L_{Y} = L_{h(Y,\;\lambda )} \times \frac{d(h(Y,\;\lambda ))}{dY} = L_{h(Y,\;\lambda )} \times Y^{\lambda - 1} $$

10 $${-} 2LL_{Y} = {-} 2LL_{h(Y,\;\lambda )} - 2(\lambda - 1)\log (Y)$$

Implementation of the dynamic estimation of λ was readily available in NONMEM VI [19] and was adapted for NONMEM 7 (Bauer, personal communication). The full dTBS approach with power parameter has been implemented in PsN [20]. PsN supplies internal files to modify the likelihood and transform the data on the fly and adapts the code in the model file to transform the predictions. The user thus only needs to provide a control file suited for modeling of untransformed data, with residual variances coded as fixed effect parameters. Further control of dTBS settings such as initial estimates of λ and ζ is possible. PsN-supplied files and an example of a modified control file can be found in Online Resources 1–3.

### Student’s t-distribution

Instead of using transformations to obtain normally distributed residuals, one can change the distributional assumption itself. In this work we investigated the use of a Student’s t-distribution, which is a symmetric distribution defined by its degree of freedom *ν*. The t-distribution approaches the normal distribution when *ν* tends towards infinity, and shows heavier and heavier tails as *ν* decreases. The likelihood of the data when assuming t-distributed residuals is displayed in Eq. 11 [21]. This approach was implemented in NONMEM by defining the probability density function corresponding to a t-distribution in the control file while using the −2LL option in $ESTIMATION. The gamma function was calculated using the Nemes approximation [22] (Eq. 12) in NONMEM 7.2 or using the built-in gamma function GAMLN in NONMEM 7.3. The lower bound for *ν* was set to 3 to guarantee full definition of the distribution and the upper bound was set to 200 which was considered the *ν* for which the t-distribution comes back to a normal distribution. An example control file using the t-distribution can be found in Online Resource 4.

11 $$L_{Y} = \frac{{\varGamma \left( {\frac{\nu + 1}{2}} \right)}}{{\varGamma \left( {\frac{\nu }{2}\sqrt {\pi \nu Var(Y)} } \right)}} \times \left( {1 + \frac{1}{v}\frac{{(Y - f(\theta ,\;x))^{2} }}{Var(Y)}} \right)^{{ - \frac{v + 1}{2}}} $$

12 $$\varGamma (X) = \sqrt {\frac{2\pi }{X}} \times \left( {\frac{1}{e}\left( {X + \frac{1}{{12X - \frac{1}{10X}}}} \right)} \right)^{X} $$

where *L*_*Y*_ is the likelihood of the data *Y*, *Γ* is the gamma function, *ν* is the degree of freedom, *f*(*θ*, *x*) is the model prediction, *Var*(*Y*) is the variance of *Y* given the model and *X* is a random variable.

### Real data examples

The dTBS and t-distribution approaches were tested separately on 10 real data examples [23–31], which comprised 7 PK and 3 PD models. Model complexity varied from simple one compartment PK models to more complex PD models and comprised additive, proportional and combined error models. Fixed log and Box–Cox transformations of the data were used in three out of 10 models. Eight examples modeled single endpoints and two examples modeled two variables simultaneously. Some models had been developed using the FO method and were adapted to be run using the FOCEI method. The available data ranged from sparse to rich, with 2–27 observations per subject. A summary of the real data examples is given in Table 1. None of the 10 models used in this work were chosen because of an indication of skewness in the residual distribution of the original model; the models using transformations did however assume skewness on the untransformed scale.

**Table 1:** Description of the 10 real data examples used to investigate the dTBS and t-distribution approaches

Model	Data type	Model type	Error model	Transformation	Number of observations	Number of subjects
ACTH/cortisol [23]	PD	Turnover	Combined^a^	–	364	7
Cladribine [24]	PK	IV 3CMT	Combined	–	488	65
Cyclophosphamide/metabolite [25]	PK	Oral 4CMT, CL induction	Additive (parent) Combined (metabolite)	–	383	14
Ethambutol [26]	PK	Oral 2CMT, transit	Combined	Log	1869	189
Moxonidine PK [27]	PK	Oral 1CMT	Additive	Log	1021	74
Moxonidine PD [27]	PD	E_max_	Additive	Log	1364	97
Paclitaxel [28]	PD	Transit	Additive	Box–Cox (*λ* = 0.2)	523	45
Pefloxacin [29]	PK	IV 1CMT	Proportional	–	337	74
Phenobarbital [30]	PK	IV 1CMT	Proportional	–	155	59
Prazosin [31]	PK	Oral 1CMT	Proportional	–	887	64

*IV* intravenous, *CMT* compartment, *CL* clearance

^a^Additive component fixed

In the dTBS approach, the Box–Cox and power parameters were estimated simultaneously using both the FOCEI and SAEM methods. Estimation of the Box–Cox parameter alone (assuming an additive RUV model on the transformed scale) and estimation of the power parameter alone (keeping the same transformation as in the original model) were also investigated using the FOCEI method. For the t-distribution approach, the scedasticity model was kept identical to the original model and the degree of freedom was estimated simultaneously to all other model parameters using the Laplacian method with user-defined likelihood.

The impact of the new error models was assessed based on diagnostic tools commonly used in NLMEM. The likelihood ratio test (LRT) was used to assess whether the proposed approaches significantly improved model fit. The LRT was based on the difference in OFV (ΔOFV) between the new and the original error model, which was assumed to follow a χ^2^ distribution. The degree of freedom of this distribution was set to the difference in the total number of estimated model parameters between these two models. The new strategies were judged to significantly improve model fit if the absolute value of ΔOFV was greater than 3.84 for the t-distribution (1 degree of freedom) and greater than 5.99 for dTBS (2 degrees of freedom). Parameter estimates and if available their SEs obtained from the asymptotic covariance matrix were contrasted. Improvements in fit were also investigated based on plots of observations versus individual predictions, individual plots and visual predictive checks. The distribution and scedasticity of conditional weighted residuals (CWRES), normalized prediction errors (NPDE) and individual weighted residuals (IWRES) were analyzed. CWRES and NPDE are not provided by NONMEM when using user-supplied likelihood and were obtained by evaluation of the model and (transformed) data at the final parameter estimates. Further investigations regarding influential individuals and predictive properties were also undertaken. Changes in individual OFVs (OFV_i_) were investigated as influence diagnostics in order to detect whether most or a subset of individuals benefited from a given residual error model structure. Cross-validation techniques using 10 splits of the data (except for the ACTH model, which was not cross-validated as it contained only seven patients) were used to assess the predictive performance of the dTBS approach.

### Simulations

Stochastic simulations and estimations (SSEs) were performed in order to investigate the estimation properties of the new error parameters in terms of bias, precision and type I error rate. The simulation model was a one-compartment disposition, first order absorption and elimination model displaying either an additive, a proportional or an additive on log scale error model. Population values used for simulation were a clearance (CL) of 10 l/h, a volume of distribution (V) of 100 l and an absorption constant (KA) of 1/h. The standard deviation (SD) of the IIV was set to 30 % for all three parameters. The RUV was set to 0.2 for the additive and additive on log models and to 20 % for the proportional model. The dataset comprised 400 observations from 50 patients from whom 8 PK samples were taken 0.25, 0.5, 1, 2, 5, 8, 12 and 24 h after administration of a single oral dose of 1000 mg. dTBS scenarios were run using both the FOCEI and SAEM estimation methods while t-distribution scenarios were run using the Laplace estimation method. All scenarios were investigated based on 500 SSE samples.

### Software

All fits were performed using NONMEM 7.2 and 7.3 [15] aided by PsN version 3.5.2 and higher [20]. Graphical output was generated with RStudio 0.98 using R 3.1.2 and lower as well as Xpose 4.3.4 and higher [32].

## Results

### Real data examples: dTBS

**Fig. 1:**
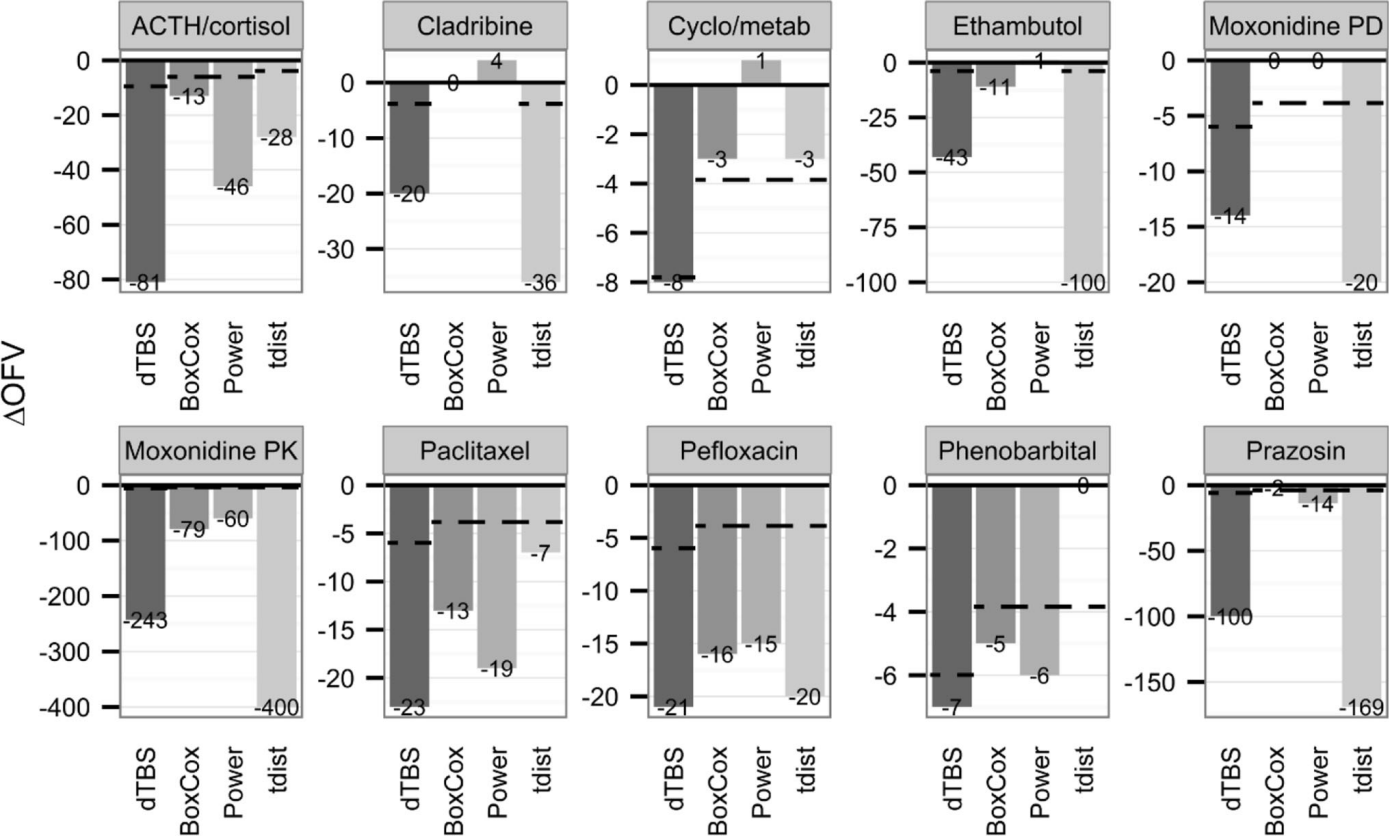
Differences in OFV (ΔOFV) between the original and the dTBS, Box–Cox, power and t-distribution models for the 10 real data examples. The *dashed lines* indicate the threshold for significant improvement over the original error model given the appropriate degree of freedom

**Fig. 2:**
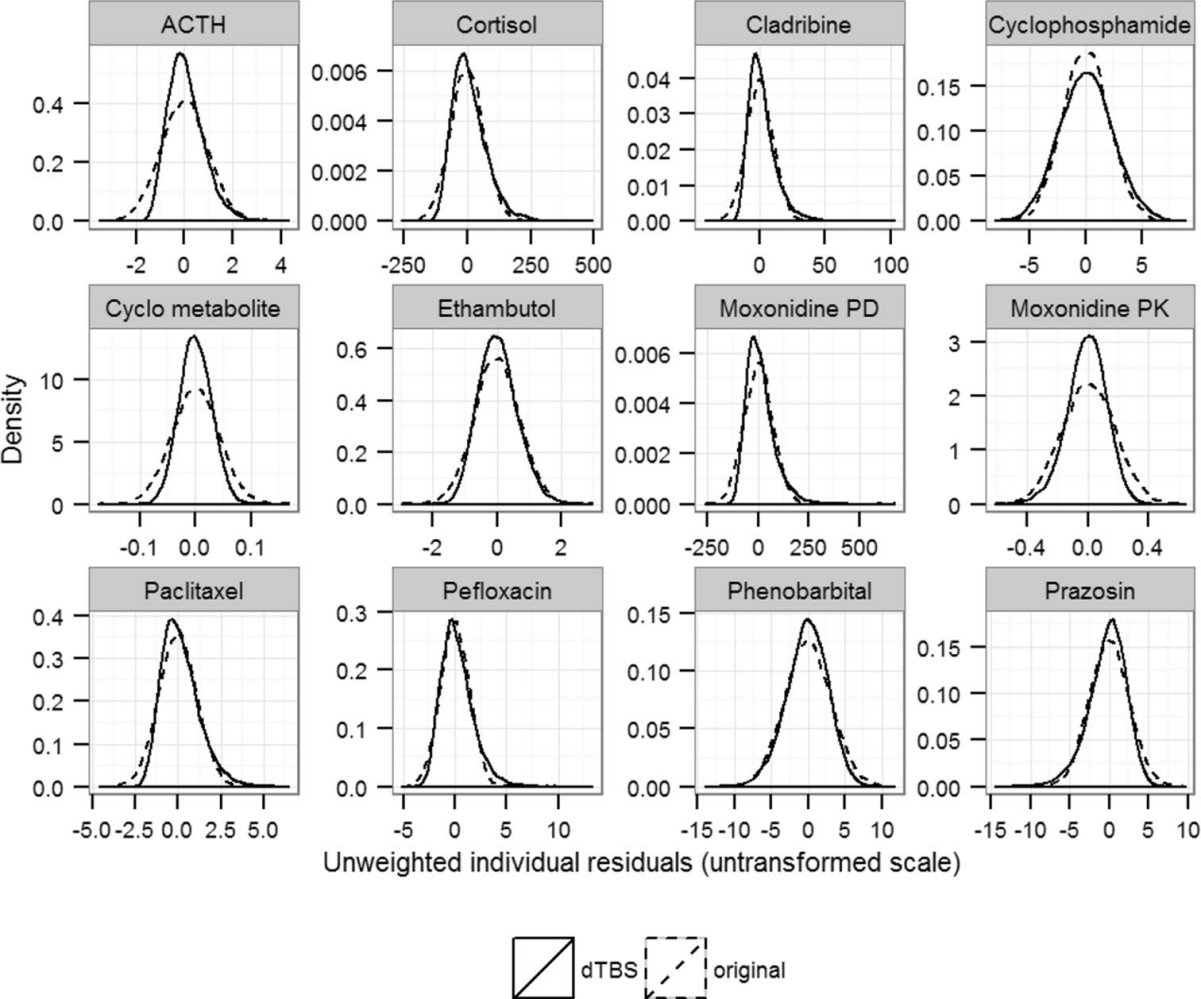
Simulated residual error distributions on the untransformed scale for the original and dTBS error models for the 12 endpoints of the 10 real data examples. *Dotted lines* correspond to the original error model and *full lines* to the dTBS error model. These distributions were obtained through simulations using the final dTBS/original estimates. The standard deviations of the distributions were calculated based on the medians of the observed data

**Fig. 3:**
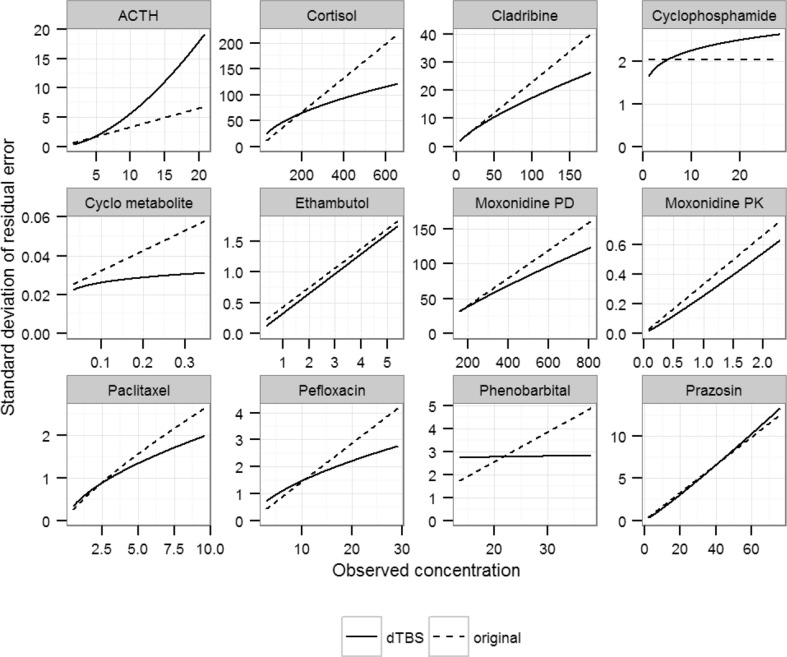
Standard deviation of the residual error variance as a function of the observed data for the original and dTBS error models for the 12 endpoints of the 10 real data examples. *Dotted lines* correspond to the original error model and *full lines* to the dTBS error model

Estimating the Box–Cox and power parameters simultaneously led to significant ΔOFV in comparison to the original model for all investigated datasets. ΔOFV were large, ranging from −243 for the moxonidine PK example to −7 for the phenobarbital example (Fig. 1). The estimated skewness λ ranged from −1 to 2.5 (Table 2). The estimated power ζ showed a similar spread. These parameters were estimated with good precision when precision was available, all models displaying low SEs except for pefloxacin (SE = 0.6) and cladribine (SE = 1). An illustration of the consequence of skewness can be found in Fig. 2, where simulated distributions of unweighted and untransformed residuals are compared between the dTBS and the original models. A comparison of the scedasticity and the size of the residual error between the dTBS and the original models can be made based on Fig. 3, which displays the absolute SD of the residual error over the range of the observed data for the 12 endpoints of the 10 real data examples. SDs using the dTBS models were lower than with the original models in 8 out of 10 cases. At the highest data point for example, the SD of the RUV using dTBS ranged between 0.54- and 2.83-fold that of the original model, with an average value of 1. On a more technical note, runtimes did not differ much between the original and dTBS models. Regarding the influence of the estimation method, skewness estimates obtained with FOCEI and SAEM were close except for the paclitaxel and pefloxacin models. In these examples, SAEM *λ* estimates confirmed the direction of the skewness found using FOCEI but indicated a higher degree of skewness (*λ* = −0.6 vs. 0.15 for paclitaxel, *λ* = −1 vs. −0.79 for pefloxacin). Approximated power on the untransformed scale stayed similar between the two estimation methods. Estimating both dTBS parameters simultaneously was significantly better than estimating only one of them for all models except phenobarbital. ΔOFV were significant for 6 out of 10 models when only the Box–Cox parameter or the power parameter were estimated. Only two examples (cladribine and cyclophosphamide/metabolite) displayed a worse model fit than with the original model when estimating a single power parameter, which was related to the fact that they were originally modelled with combined error models. Estimates of *λ* obtained using a fixed additive error model on the transformed scale differed from those obtained using dTBS. However, the direction of the estimated skewness stayed identical except for moxonidine PK and prazosin, whose estimated *λ* values indicated left-skewness using dTBS but right-skewness using the Box–Cox alone. The estimated power ζ also differed depending on whether it was estimated alone or together with *λ*. For most models, these changes however translated into similar scedasticity, as evidenced by small differences between approximated powers on the untransformed scale when using the dTBS, Box–Cox and power error models (Online Resource 5). These results confirmed that only the combined approach could correct simultaneously for skewness and scedasticity and thus the Box–Cox or power error models alone were not further investigated in this work.

**Table 2:** Estimated error parameters, associated standard errors (SEs) and ΔOFV using the dTBS and the t-distribution approaches for the 10 real data examples

Model	Original error model	ε-Shrinkage (%)	dTBS^b^				t-Distribution	
			*λ* (SE)	*ζ* (SE)	Approximated scedasticity 1 − *λ* + *ζ*	ΔOFV	*ν*	ΔOFV
ACTH/cortisol [23]	Combined^a^	2.9	0 (–)	0.68 (0.27)	1.68	−86	3	−28
			0 (–)	−0.47 (0.18)	0.53			
Cladribine [24]	Combined	15.8	−0.65 (1.2)	−0.92 (1.0)	−0.58	−20	5	−36^c^
Cyclophosphamide/metabolite [25]	Additive (parent)	5.4	0.85 (–)	0 (–)	0.15	−8.6	9	−2.6
	Combined (metabolite)		0.86 (–)	0 (–)	0.16			
Ethambutol [26]	Combined on log	11.8	0.67 (0.21)	0.67 (0.16)	1	−43	3	−100^c^
Moxonidine PK [27]	Additive on log	11.6	1.5 (0.066)	1.6 (0.076)	1.1	−243	3	−400
Moxonidine PD [27]	Additive on log	11.4	−0.93 (–)	−1.1 (–)	0.84	−14	9	−25
Paclitaxel [28]	Additive on Box–Cox	19.3	0.15 (–)	−0.25 (–)	0.6	−22	3	−7.4^c^
Pefloxacin [29]	Proportional	23.2	−0.79 (0.61)	−1.2 (0.58)	0.59	−21	4.7	−20
Phenobarbital [30]	Proportional	28.9	1.8 (0.44)	0.83 (0.23)	0.03	−7	∞	0
Prazosin [31]	Proportional	11.2	2.4 (0.17)	2.5 (0.16)	1.1	−100	3	−169

^a^Additive component fixed

^b^Presented dTBS results are those obtained with the FOCEI method

^c^Standard estimation of ν impossible, estimated through likelihood profiling

Estimates of non-residual error model parameters and related precision using the dTBS approach differed from those obtained using the original models in a number of cases. Table 3 presents examples of such changes. Some models presented changes in both selected fixed effects and random effects variances, some presented changes only in random effects and others displayed no changes at all in population parameters. Changes in parameter estimates were deemed physiologically plausible based on the available insight on the model and data. dTBS also impacted the precision of parameter estimates, which could be improved or deteriorated depending on the model and parameter.

**Table 3:** Selected examples of changes in non-residual error model parameters when the dTBS and the t-distribution approaches are used

Model	Changes in non-residual error model parameters with dTBS	Changes in non-residual error model parameters with the t-distribution
ACTH/cortisol [23]	4-fold decrease in surge amplitude 1.5-fold increase in maximum effect 2-fold increase in concentration leading to half the maximum effect 2-fold decrease in Hill coefficient	25 % reduction in surge amplitude Modification of the models parameters related to the E_max_ function
Cladribine [24]	Unchanged estimates Higher RSE	15 % decrease in inter-compartmental clearance 50 % increase in IIV of volume of distribution
Cyclophosphamide/metabolite [25]	Ratio between induced and non-induced clearance decreases from 5 to 1 1.5-fold increase in maximum effect	Limited changes in estimates^a^
Ethambutol [26]	20–30 % change in volumes of distribution, mean transit time, absorption rate and related IIV	20–30 % change in volumes of distribution, mean transit time, absorption rate and all IIV
Moxonidine PK [27]	1.2-fold increase in IOV of absorption rate 0.8-fold decrease in IIV of absorption rate	3-fold decrease in IIV of absorption rate
Moxonidine PD [27]	1.5-fold increase of maximum effect 0.3-fold decrease in transfer rate constant to the effect compartment	1.3-fold increase in transfer rate constant to the effect compartment
Phenobarbital [30]	IIV of clearance increased from 33 to 44 %, lower uncertainty (RSE 22 vs. 63 %)	*no change as ν* = *∞*
Pefloxacin [29]	IOV of volume decreased from 9 to 4.7 %, higher uncertainty (RSE 97 vs. 42 %)	Unchanged estimates
Prazosin [31]	Unchanged estimates Unchanged RSE	50 % increase in covariate effect of race on clearance

*IIV* inter-individual variability, *IOV* inter-occasion variability, *RSE* relative standard error

^a^Not discussed since standard estimation of ν impossible, see “Results” section for the t-distribution

**Fig. 4:**
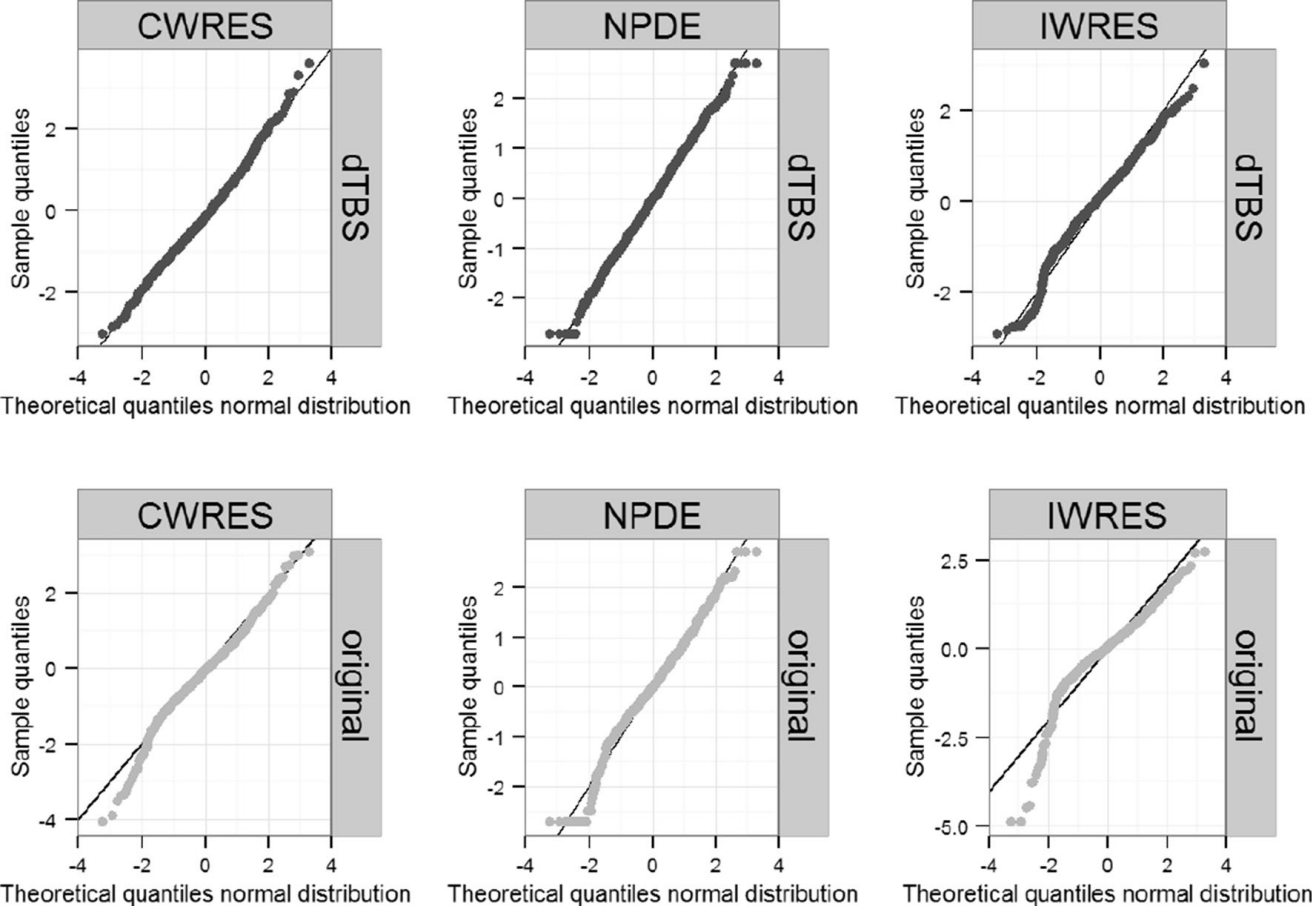
CWRES, NPDE and IWRES QQ-plots for the original and dTBS error models in the prazosin example. *Dark circles* correspond to the final dTBS model, *light circles* to the original model. Sample quantiles are compared to the theoretical quantiles of a standard normal distribution

Changes in plots of observations versus individual predictions, individual plots and visual predictive checks were typically minor (data not shown). Residual distributions of CWRES, NPDE and IWRES showed some improvement for examples with high ΔOFV such as moxonidine PK, ethambutol and prazosin (Fig. 4) but little difference in other cases.

### Real data examples: Student’s t-distribution

Using a t-distribution with estimated degrees of freedom led to significant improvement in ΔOFV for five out of the seven models for which the estimation of the degree of freedom was successful. The cyclophosphamide/metabolite and phenobarbital examples did not benefit from t-distributed residuals (Fig. 1; Table 2). When significant, ΔOFV were large, ranging from −400 for the moxonidine PK example to −20 for the pefloxacin example. Estimated *ν* values spanned the entire range of possible *ν* values (from *ν* = 3 to 200) with point estimates between 3 and 9 for significantly improved models (Table 2). The moxonidine PK, prazosin and ACTH/cortisol examples showed the heaviest tails, whereas the estimated degree of freedom in the phenobarbital example pointed toward normally distributed residuals. Asymptotic SEs on *ν* could not be estimated either because of model instability or because *ν* was estimated at its upper or lower boundary. It should be noted that the use of the Laplacian method in NONMEM (instead of the FO or FOCE methods on which the models were originally developed) often led to minimization problems and a failure of the covariance step, and this regardless of which distribution was used. In the cladribine, ethambutol and paclitaxel examples, *ν* stayed around any given initial guesses but changes in OFV were nevertheless observed when fixing *ν* to different values. Models for which estimation of *ν* was not possible will not be discussed here.

**Fig. 5:**
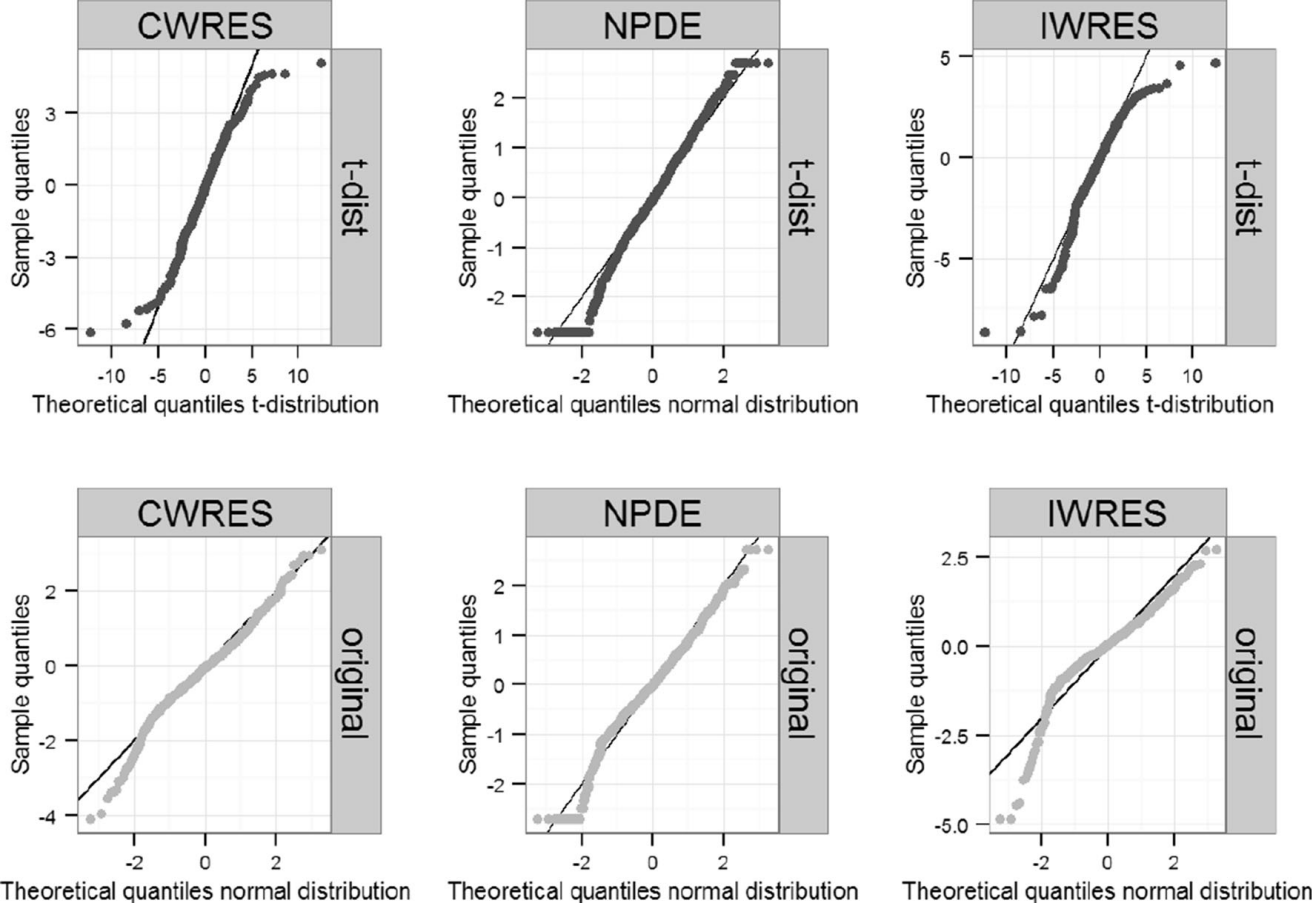
CWRES, NPDE and IWRES QQ-plots for the original and t-distributed error models in the prazosin example. *Dark circles* correspond to the final t-distribution model, *light circles* to the original model. Sample quantiles are compared to the theoretical quantiles of a standard normal distribution for the original model and to that of a standard normal distribution (NPDE) or a t-distribution with 3 degrees of freedom (CWRES, IWRES) for the t-distributed error model

As with the dTBS approach, physiologically plausible changes in estimates of non-residual error model parameters using the t-distribution approach were observed (Table 3). Individual predictions in the moxonidine example evidenced a better agreement to the observations by being further away from a limited number of outliers. Consequences of changes in estimated parameters on individual predictions were less apparent for the other examples. As observed with the dTBS approach, changes in the agreement between observed and expected residual distributions could be observed (Fig. 5) for some but not all models.

### Simulations: dTBS

Simulated additive, proportional and additive on log error models could be re-estimated using dTBS (Table 4). *λ* estimates were unbiased with the additive on log error model error model but showed downward bias of 0.13 and 0.26 for the additive and proportional error models. Using SAEM instead of FOCEI removed the bias on *λ* for the proportional error model. Estimates of δ (ζ was reparameterized in the simulation exercise) were unbiased even in the presence of bias in *λ*. The SEs of dTBS parameters were small (below 0.25) for the proportional and additive on log models. Precision on *λ* was poor (SE = 0.73) for the additive model. Type I error was controlled at the investigated dataset size for the additive and additive on log error models when using FOCEI and for the proportional error model when using SAEM. Other model parameter estimates and related precision were similar whether dTBS parameters were estimated or fixed to their true values.

**Table 4:** Estimated bias, precision and type I error rates for the dTBS error model and type I error rates for the t-distribution error model in the simulation examples (500 SSE samples)

Error models	Estimation methods	dTBS							t-Distribution
		True *λ*	True *δ*	Bias *λ*	Bias *δ*	SD *λ*	SD *δ*	Type I error (%)	Type I error (%)
Additive	FOCEI	1	−1	−0.13	−0.084	0.73	0.10	2.00	0
Proportional	FOCEI	1	0	−0.26	−0.020	0.26	0.088	11.20	0
Proportional	SAEM	1	0	−0.036	0.033	0.31	0.092	4.40	–
Additive on log	FOCEI	0	0	−0.022	0.0066	0.24	0.087	3.60	0.20

### Simulations: t-distribution

Additive, proportional and additive on log scale error models simulated under the normality assumption could be re-estimated using the t-distribution. The estimates of the degrees of freedom tended towards the given upper bound of 200. Type I error was close to 0 % for all simulated error models.

## Discussion

Implementation of both the dTBS and the t-distribution approaches in NONMEM was successful apart from stability issues related to the use of the Laplacian method with some of the investigated models. The additional error parameters could be estimated using ML and OFV could be compared to select the most appropriate model. The new error models improved model fit for the investigated real data examples.

### Real data examples: dTBS

Significant OFV drops were observed for all models when allowing for skewness in the residual error. OFV was expected to be sensitive to changes in the residual error, and power to detect such changes is expected to be high as all observations contribute to the determination of the residual error model.

Point estimates of *λ* indicated some degree of skewness in the distribution of the residuals for all models. Absence of skewness could not be excluded for the phenobarbital and cladribine examples, for which asymptotic 95 % confidence intervals on *λ* included the reference value of 1. The estimated shape parameter indicated right-skewed residuals (i.e., higher positive than negative residuals) on the untransformed scale for 7 out of 10 models. This is in line with the type of data used here, where the presence of left-censoring as a result of the impossibility to observe negative endpoint values often leads to right-skewness of the residuals. Two PK examples with rich sampling showed left-skewness (moxonidine PK and prazosin) possibly as a consequence of absorption model misspecifications. Box–Cox estimates for variables which were simultaneously modeled (ACTH/cortisol and cyclophosphamide/metabolite) stayed similar, which was not unexpected since both profiles and assays were homogenous in the studied cases.

The shape parameter *λ* differed greatly depending on whether the power parameter ζ was estimated or fixed to 0 as in the traditional TBS approach. This could be explained by the fact that in the latter case, *λ* determines not only the skewness but also the scedasticity, which is then equal to the power of (1 − *λ*). It appeared that estimating *λ* alone was correcting for scedasticity more than for skewness, which makes sense as scedasticity is expected to have a much higher impact on model fit than skewness. This was best illustrated in the paclitaxel example, originally modelled with a Box–Cox transformation of 0.2 corresponding to a scedasticity of 0.8 on the untransformed scale (*λ* = 0.2, δ = 0.8). Using dTBS, both the right-skewness and the lower than proportional scedasticity were confirmed but were free to take on slightly lower values (*λ* = 0.15, δ = 0.6). It follows that to correct for both skewness and scedasticity, one should use the full dTBS approach and not the Box–Cox transformation alone. Regarding the value of *λ* itself, skewness is most likely multifactorial and depends on a mixture of endpoint type (presence of physiological boundaries for example), study design (inclusion/exclusion criteria), assay characteristics, and model misspecification. Identification and categorization of causes leading to the presence of skewed residuals by data and/or model types is not straightforward and as such it will be difficult to anticipate the likely value of *λ* in a given setting.

The approximated power on the untransformed scale was estimated to be proportional (four models), close to additive (two models) or somewhere in between (three models). The ACTH/cortisol model showed the highest scedasticity, with an error approximately proportional to the square of the ACTH predictions, which may be explained by the dichotomous nature of this endpoint, with a cluster of values very close to 5 and the other above 15. There was no clear trend between original and dTBS scedasticity, with some models keeping their original scedasticity (moxonidine PK and PD, prazosin) and others differing. Phenobarbital was however the only model for which the additive error model was found to fit the data better than the original proportional model. This had been concluded previously [33] and is to relate to the small data range investigated, which confounds the discrimination between additive and proportional error models. A constant variance, or even a second power term, could in theory be added to the dTBS model. However, while combined error models make sense on the untransformed scale, they translate into “double” power models when used in combination with a transformation, which may be both hard to estimate and to interpret. In addition, it was believed that the combination of power and Box–Cox would be flexible enough to describe observed residual error patterns, which was supported by the fact that the addition of a second ζ term for the combined error models did not improve model fit (data not shown).

As already observed for individual parameter distributions [34], better agreement of the distribution of residuals to the normality assumption was difficult to assess visually. The observed IWRES distributions could be confounded by the presence of high ε-shrinkage (mostly between 10 and 15 % but up to 20–30 % for paclitaxel, pefloxacin and phenobarbital), which renders the interpretation this diagnostic subject to caution. Other residuals such as CWRES and NPDE, which are not sensitive to shrinkage, were available. However, these metrics are affected by all random effects of the model (IIV, IOV and RUV) and thus are less specific to the residual error. These observations highlight limitations of standard residual error diagnostics in NLMEM.

Visualizing the impact of changes in other model parameter estimates in order to diagnose the RUV model did not prove more supportive than residual diagnostics, which was not unexpected since the impact of changes in the RUV model should be minor compared to changes in the structural model. However, estimating dTBS error parameters could lead to changes at all levels of NLMEM: estimates of fixed and random effects as well as related precision. Part of the difficulty in interpretation is due to the interaction between the different levels of variability in NLMEM.

Limitations of commonly used goodness-of-fit diagnostics drew focus on the OFV as the main criterion to indicate improvement of the residual error model. To complement this criterion, additional investigations using influence diagnostics and predictive properties were conducted in this work. Changes in individual OFV were used to investigate the influence of individuals on dTBS parameters (Online Resource 6). The dTBS approach always benefitted the majority of individuals in a given dataset, with an average proportion of individuals improved of 64 % (range [51–100 %]) over the 10 models. The size of the overall OFV drop was related to the number of individuals who benefited from dTBS, the highest ΔOFVs being observed in examples for which dTBS was highly beneficial for many individuals. The proportion of individuals responsible for the significant part of the ΔOFV was 14 % on average. This may appear low, but it is important to note that the very nature of distributions such as the Box–Cox predisposes it to individual influence, as only a limited part of the distribution really deviates from a normal distribution. The superiority of the dTBS model when taking all individuals into account was confirmed by better predictive properties as assessed through cross-validation. The OFV sum over cross-validation datasets was lower with dTBS than with the original models for all examples except cladribine and pefloxacin, and dTBS parameter estimates were consistent between training datasets (data not shown). The two examples that showed worse predictive properties and inconsistent estimates (λ = −1.6 instead of −0.7 for cladribine, λ = 2 instead of −0.8 for pefloxacin) also displayed high SEs on dTBS parameters, which would discourage the use of the dTBS error model. To summarize, this indicated that accounting for deviations, even if they are small and localized, is beneficial on the group level, and it is known that a limited number of outliers can overwhelm ML estimation [16]. The investigation of influential individuals also confirmed the interaction between the different levels of variability in NLMEM. Indeed, the individuals who supported most of the ΔOFV were the ones displaying the greatest changes in empirical Bayes estimates, as seen in the cladribine, cyclophosphamide/metabolite and ethambutol examples. Note that individual influence was less marked for the power parameter, which by definition has a smaller tendency of being outlier-driven. The inclusion of an estimated power of the mean error parameter was shown to be robust to the skewness model, with the approximated power on the untransformed scale staying fairly constant over various values of the shape parameter. The incorporation of IIV on error parameters was not considered here but could be envisaged if the number of observations per individual is high enough for this to be estimable.

### Real data examples: t-distribution

The 10 investigated real data examples benefitted from various degrees of heavy tails. Examples with highest ΔOFV often had an estimated degree of freedom of 3, and all models showing a significant improvement had a *ν* below 10. As with the dTBS approach, reasons to observe heavy tails in the distribution of the data are most likely multifactorial, and the anticipation of the value of *ν* in a given setting is difficult.

Residual diagnostics faced the same limitations as with dTBS, namely the lack of specificity and the sensitivity to ε-shrinkage. Changes in residual plots were not always visible but in general more pronounced than with dTBS. This could be expected as the t-distribution allows more extreme residuals (and this more frequently) than the Box–Cox transformation. Changes in goodness-of-fit plots based on individual predictions were also not always straightforward to detect, but in some cases such as moxonidine PK an overall better agreement was seen at the cost of few outlying data points.

Individual influence was also investigated for the t-distribution, even if it was not unexpected when using such distributions. The conveyed picture was similar to that observed with the dTBS approach. The average proportion of individuals improved when the t-distribution lead to a significant ΔOFV was 71 % (range [51–86 %]) over five models, with a proportion of individuals responsible for the significant part of the ΔOFV of 29 % on average. Predictive properties using cross-validation were not investigated for the t-distribution due to the instability of the investigated models with the Laplacian method.

The incorporation of IIV in RUV, either as a continuous distribution [35] or a mixture model with two different residual error variances allowing an estimated fraction of the individuals to display higher RUV than the rest of the individuals, was not investigated here but could also be considered. Other distributions could also be considered: mixture models at the observation level, power exponential models, Cauchy, Laplace, Gamma and Weibull have previously been used in the PK literature [13, 36] and have shown improvement over the normality assumption. It should be noted that current implementation using the Laplacian method proved limiting in this case, as it led to minimization difficulties for both the normal and Student’s probability distribution functions in about half of the models. This should be addressed to guarantee efficient use of the t-distribution and potentially other distributions.

### Simulation examples

The design investigated here presented rich sampling at the individual level but a moderate number of total observations as compared to the real data examples. The downward bias of −25 % in *λ* despite low SE led to a doubling of the type I error with the FOCEI method when an interaction between predictions and residuals was present. This was corrected when using the SAEM method, hinting towards limitations of the FOCEI method in the presence of scedasticity and high nonlinearity. Bias of −10 % on *λ* in the additive model did not lead to an increase in type I error, probably due to the high SE observed for this parameter. Part of this bias could be linked to the censoring of negative concentrations (around 1 % of simulated concentrations per dataset), which may have induced some right-skewness in the simulated data. The high uncertainty on *λ* could be linked to the relatively low variance used for this model, as SE on *λ* decreased to levels similar to those observed for the proportional and additive on log models when residual variance was increased (data not shown). Estimates and precision of parameters not related to the residual error were consistent across all error models in the simulated settings.

### Comparison of dTBS and t-distribution approaches

Interestingly, dTBS and t-distribution ΔOFV were often similar and real data examples truly benefitting from the two approaches were the same. This could at first seem counterintuitive as the two error models have conflicting assumptions regarding the presence of skewness. However, the dTBS and t-distribution approaches are similar in that they both allow some individual predictions to be further away from the observations. The difference lies in the balance (t-distribution) or the mismatch (dTBS) between the numbers of positive and negative high residuals, thus the presence or absence of skewness. One can easily imagine that if uni- or bilateral outlier observations are present, an approach that allows some type of outliers will be beneficial even if the symmetry is misspecified. Another difference between the two approaches lies in the number of outlying residuals they allow, and more generally where the mass of the residuals is expected to be. For example, a t-distribution with *ν* = 3 allows 14.5 % of the residuals to have an absolute value greater than 1.96, compared to 2.8 % with a dTBS distribution with *λ* = 2 and 5 % for the normal distribution, given a SD of 1 for all. Models with more extreme outliers may thus benefit from a t-distribution more than they do from dTBS even if these outliers are not symmetric, as was seen in the moxonidine PK and prazosin examples. Overall, both approaches have the advantage of being capable of handling potential outliers while avoiding subjective predefined exclusion criteria or case-by-case handling of outlying data points. It can also be noted that both approaches, in particular the t-distribution, address kurtosis and can thus correct both for peaked and/or heavy-tailed distributions. When using these approaches, one naturally needs to ensure the absence of trends in the estimated skewness or outlying data points to avoid any masking of potential model misspecification by the residual error model, particularly during model building. However, once a model is deemed as good as it can be, handling often inevitable remaining model misspecification through the use of one of the proposed approaches is more indicated than simply ignoring it. The two approaches could also be combined, allowing for both heavy tails and skewness. This would also permit to treat dTBS and the t-distribution as nested models and thus to select the most beneficial one based on truly comparable ΔOFV. This was done using the prazosin example (model code provided in Online Resource 7). In this example, no skewness was detected when estimating *ν*, *λ* and *ζ* simultaneously, confirming that the t-distribution was more beneficial. However, as for any component of a model, any added complexity always needs to be balanced with potential gains, which may be more limited for the residual error than for other components of the model.

### How to proceed in practice

When to use the proposed approaches is naturally a relevant question. The choice between these approaches can be guided by the presence or absence of skewness in the residual distribution. If a final model is obtained through traditional model building, it is recommended to apply dTBS and/or t-distributions as a mean to improve the robustness of conclusions drawn from the model. However, these approaches could also be introduced earlier in the model building, when choices between error models are usually made and when diagnostics indicate deviations from standard residual error assumptions. If they appear beneficial, they should be retained. If possible, RUV parameters should not be fixed during model building to maintain flexibility with regards to changes in other, more important parts of the model.

## Conclusion

The dTBS and Student’s t-distribution approaches are two approaches capable of addressing non-normal residuals. The dTBS approach is able to adjust both skewness and scedasticity while the t-distribution allows for symmetric heavy tails in the residual distribution. Both approaches can be combined in a general and flexible framework which addresses the trial-and-error strategy usually employed for residual error model building. While getting the statistical model right is not a primary aim, it is believed that scrutiny towards residual error assumptions will enhance the quality of model-based analysis and any subsequent simulations.

## Electronic supplementary material

Supplementary material 1 (DOCX 37 kb)

Supplementary material 2 (DOCX 16 kb)

Supplementary material 3 (DOCX 15 kb)

Supplementary material 4 (DOCX 17 kb)

Supplementary material 5 (DOCX 24 kb)

Supplementary material 6 (DOCX 238 kb)

Supplementary material 7 (DOCX 17 kb)
