# Supplementary material for: A strategy for residual error modeling incorporating scedasticity of variance and distribution shape
Source: J Pharmacokinet Pharmacodyn. 2015 Dec 17;43:137–51. doi: 10.1007/s10928-015-9460-y (PMC4791481; doi:10.1007/s10928-015-9460-y)
Supplement: Supplementary file 1 — Supplementary material 1 (DOCX 37 kb) [file 10928_2015_9460_MOESM1_ESM.docx]

## **Online Resource 1: dTBS model file (phenobarbital example)**

| Article title | A Strategy for Residual Error Modeling Incorporating Scedasticity of Variance and Distribution Shape |
| --- | --- |
| Journal name | Journal of Pharmacokinetics and Pharmacodynamics |
| Author names | Anne-Gaëlle Dosne^1^, Martin Bergstrand^1^, Mats O Karlsson^1^ |
| Author affiliations | ^1^Department of Pharmaceutical Biosciences, Uppsala University, P.O. Box 591, 751 24 Uppsala, Sweden |
| Corresponding author | Anne-Gaëlle Dosne: [annegaelle.dosne@farmbio.uu.se](mailto:annegaelle.dosne@farmbio.uu.se) |

**Caption**: Example NONMEM model file using dTBS. Here the dTBS model is hard-coded within the model file. It corresponds to what PsN would produce based on a model file that does not include dTBS.

$PROBLEM PHENOBARB model

;; dTBS model

$INPUT ID TIME AMT WT APGR DV

$DATA PHENO.dta IGNORE=@

$SUBROUTINE ADVAN1 TRANS2 CONTR=../../contr.txt CCONTR=../../ccontra_nm7.txt

$PK

TVCL = THETA(1)

CL = TVCL*EXP(ETA(1))

TVV = THETA(2)

V = TVV*EXP(ETA(2))

S1 = V

$ERROR

IPR1 = F

IF(F.LE.0) IPR1 = 0.001

;-------------- dTBS ------------------------------

LAMBDA = THETA(5) ; Box-Cox parameter

ZETA = LAMBDA + THETA(4) ; power

W = THETA(3)*IPR1**ZETA

IPRED = IPR1

IPRTR=IPRED

IF (LAMBDA .NE. 0 .AND. IPRED .NE.0) THEN

IPRTR=(IPRED**LAMBDA-1)/LAMBDA

ENDIF

IF (LAMBDA .EQ. 0 .AND. IPRED .NE.0) THEN

IPRTR=LOG(IPRED)

ENDIF

IF (LAMBDA .NE. 0 .AND. IPRED .EQ.0) THEN

IPRTR=-1/LAMBDA

ENDIF

IF (LAMBDA .EQ. 0 .AND. IPRED .EQ.0) THEN

IPRTR=-1000000000

ENDIF

IPRED=IPRTR

IRES = DV - IPRED

IWRES = IRES/W

IWRTR=IWRES

IF (LAMBDA.NE.0 .AND. DV.NE.0 .AND. W.NE.0) THEN

IWRTR=((DV**LAMBDA-1)/LAMBDA-IPRED)/W

ENDIF

IF (LAMBDA.EQ.0 .AND. DV.NE.0 .AND. W.NE.0) THEN

IWRTR=(LOG(DV)-IPRED)/W

ENDIF

IF (LAMBDA.NE.0 .AND. DV.EQ.0 .AND. W.NE.0) THEN

IWRTR=(-1/LAMBDA-IPRED)/W

ENDIF

IF (LAMBDA.EQ.0 .AND. DV.EQ.0 .AND. W.NE.0) THEN

IWRTR=(-1000000000-IPRED)/W

ENDIF

IWRES=IWRTR

Y=IPRED+EPS(1)*W

IF (ICALL.EQ.4 .AND. LAMBDA.EQ.0) THEN

Y=EXP(Y)

ENDIF

IF (ICALL.EQ.4 .AND. LAMBDA.NE.0) THEN

Y=((Y*LAMBDA)+1)**(1/LAMBDA)

ENDIF

$THETA (0,.0059) ; 1. TVCL

$THETA (0,1.45) ; 2. TVV

$THETA (0, 2.53) ; 3. RV

$THETA (-2,0.001,2) ; 4. DELTA_ZETA

$THETA 0.831 ; 5. LAMDA

$OMEGA 0.194 ; 1. variance for ETA(1)

$OMEGA 0.201 ; 2. variance for ETA(2)

$SIGMA 1 FIX

$ESTIMATION METHOD=1 INTER MAXEVAL=9999

$COV
