# Supplementary material for: A strategy for residual error modeling incorporating scedasticity of variance and distribution shape
Source: J Pharmacokinet Pharmacodyn. 2015 Dec 17;43:137–51. doi: 10.1007/s10928-015-9460-y (PMC4791481; doi:10.1007/s10928-015-9460-y)
Supplement: Supplementary file 2 — Supplementary material 2 (DOCX 16 kb) [file 10928_2015_9460_MOESM2_ESM.docx]

## **Online Resource 2: dTBS ccontra file (phenobarbital example)**

| Article title | A Strategy for Residual Error Modeling Incorporating Scedasticity of Variance and Distribution Shape |
| --- | --- |
| Journal name | Journal of Pharmacokinetics and Pharmacodynamics |
| Author names | Anne-Gaëlle Dosne^1^, Martin Bergstrand^1^, Mats O Karlsson^1^ |
| Author affiliations | ^1^Department of Pharmaceutical Biosciences, Uppsala University, P.O. Box 591, 751 24 Uppsala, Sweden |
| Corresponding author | Anne-Gaëlle Dosne: [annegaelle.dosne@farmbio.uu.se](mailto:annegaelle.dosne@farmbio.uu.se) |

**Caption**: ccontra.txt file needed for NONMEM to use dTBS. This file enables the on-the-fly transformation of the observations and the calculation of the likelihood of the untransformed data. PsN writes this file and automatically takes care of the referencing of the Box-Cox parameter lambda to the corresponding THETA in the model file.

dTBS.subroutine ccontr (icall,c1,c2,c3,ier1,ier2)

USE ROCM_REAL, ONLY: theta=>THETAC,y=>DV_ITM2

USE NM_INTERFACE,ONLY: CELS

! parameter (lth=40,lvr=30,no=50)

! common /rocm0/ theta (lth)

! common /rocm4/ y

! double precision c1,c2,c3,theta,y,w,one,two

double precision c1,c2,c3,w,one,two

dimension c2(:),c3(:,:)

data one,two/1.,2./

if (icall.le.1) return

w=y(1)

if(theta(7).eq.0) y(1)=log(y(1))

if(theta(7).ne.0) y(1)=(y(1)**theta(7)-one)/theta(7)

call cels (c1,c2,c3,ier1,ier2)

y(1)=w

c1=c1-two*(theta(7)-one)*log(y(1))

return

end
