# Supplementary material for: A strategy for residual error modeling incorporating scedasticity of variance and distribution shape
Source: J Pharmacokinet Pharmacodyn. 2015 Dec 17;43:137–51. doi: 10.1007/s10928-015-9460-y (PMC4791481; doi:10.1007/s10928-015-9460-y)
Supplement: Supplementary file 3 — Supplementary material 3 (DOCX 15 kb) [file 10928_2015_9460_MOESM3_ESM.docx]

## **Online Resource 3: dTBS contr file (invariant)**

| Article title | A Strategy for Residual Error Modeling Incorporating Scedasticity of Variance and Distribution Shape |
| --- | --- |
| Journal name | Journal of Pharmacokinetics and Pharmacodynamics |
| Author names | Anne-Gaëlle Dosne^1^, Martin Bergstrand^1^, Mats O Karlsson^1^ |
| Author affiliations | ^1^Department of Pharmaceutical Biosciences, Uppsala University, P.O. Box 591, 751 24 Uppsala, Sweden |
| Corresponding author | Anne-Gaëlle Dosne: [annegaelle.dosne@farmbio.uu.se](mailto:annegaelle.dosne@farmbio.uu.se) |

**Caption**: contr.txt file needed for NONMEM to use dTBS. PsN writes this file, which stays identical whichever model is used.

subroutine contr (icall,cnt,ier1,ier2)

double precision cnt

call ncontr (cnt,ier1,ier2,l2r)

return

end
