# Supplementary material for: A strategy for residual error modeling incorporating scedasticity of variance and distribution shape
Source: J Pharmacokinet Pharmacodyn. 2015 Dec 17;43:137–51. doi: 10.1007/s10928-015-9460-y (PMC4791481; doi:10.1007/s10928-015-9460-y)
Supplement: Supplementary file 4 — Supplementary material 4 (DOCX 17 kb) [file 10928_2015_9460_MOESM4_ESM.docx]

## **Online Resource 4: t-distribution model file (phenobarbital example)**

| Article title | A Strategy for Residual Error Modeling Incorporating Scedasticity of Variance and Distribution Shape |
| --- | --- |
| Journal name | Journal of Pharmacokinetics and Pharmacodynamics |
| Author names | Anne-Gaëlle Dosne^1^, Martin Bergstrand^1^, Mats O Karlsson^1^ |
| Author affiliations | ^1^Department of Pharmaceutical Biosciences, Uppsala University, P.O. Box 591, 751 24 Uppsala, Sweden |
| Corresponding author | Anne-Gaëlle Dosne: [annegaelle.dosne@farmbio.uu.se](mailto:annegaelle.dosne@farmbio.uu.se) |

## **Caption :** Example NONMEM model file using a t-distribution for the residual error. The probability density function of the t-distribution is hard-coded within the model file, and the Laplacian method is used for estimation.

$PROBLEM PHENOBARB model

;; Base model for Phenobarbital (prop RUV on DV)

;; Student distribution DF=est

$INPUT ID TIME AMT WT APGR DV

$DATA PHENO.dta IGNORE=@

$SUBROUTINE ADVAN1 TRANS2

$PK

TVCL = THETA(1)

CL = TVCL*EXP(ETA(1))

TVV = THETA(2)

V = TVV*EXP(ETA(2))

S1 = V

$ERROR

IPRED= F

IF(F.LE.0) IPR1 = 0.001

W = THETA(3)*IPRED ; proportional residual error

DF = THETA(4) ; degrees of freedom of Student distribution

SIG1 = W ; scaling factor for standard deviation of RUV

IWRES=(DV-IPRED)/SIG1

PHI=(DF+1)/2 ; Nemes approximation of gamma funtion (gamma((DF+1)/2))

INN=PHI+1/(12*PHI-1/(10*PHI))

GAMMA=SQRT(2*3.14159265/PHI)*(INN/EXP(1))**PHI

PHI2=DF/2

INN2=PHI2+1/(12*PHI2-1/(10*PHI2))

GAMMA2=SQRT(2*3.14159265/PHI2)*(INN2/EXP(1))**PHI2

COEFF=GAMMA/(GAMMA2*SQRT(DF*3.14159265))/SIG1

BASE=1+IWRES*IWRES/DF

IF(BASE.EQ.0) BASE=0.000001

POW=-(DF+1)/2

L=COEFF*BASE**POW ; PDF of t-distribution

Y=-2*LOG(L)

$THETA (0,.005) ; 1. TVCL

$THETA (0,1.45) ; 2. TVV

$THETA (0, 0.2) ; 3. RV

$THETA (2,50,300); 4. DF

$OMEGA 0.228 ; 1. variance for ETA(1)

$OMEGA 0.146 ; 2. variance for ETA(2)

$ESTIMATION MAXEVAL=9990 -2LL METH=1 LAPLACE PRINT=10 MSFO=msf

$COV

$TABLE ID TIME IPRED IWRES CWRES NOPRINT FILE=sdtab2

$TABLE ID ETA1 ETA2 NOPRINT FILE=patab2

$TABLE ID WT APGR NOPRINT FILE=cotab2
