# Supplementary material for: A strategy for residual error modeling incorporating scedasticity of variance and distribution shape
Source: J Pharmacokinet Pharmacodyn. 2015 Dec 17;43:137–51. doi: 10.1007/s10928-015-9460-y (PMC4791481; doi:10.1007/s10928-015-9460-y)

## **Online Resource 5: Approximated scedasticity on the untransformed scale for the 12 modelled compounds of the 10 real data examples when skewness and scedasticity parameters are estimated separately (Box-Cox and Power) or simultaneously (dTBS).**

| Article title | A Strategy for Residual Error Modeling Incorporating Scedasticity of Variance and Distribution Shape |
| --- | --- |
| Journal name | Journal of Pharmacokinetics and Pharmacodynamics |
| Author names | Anne-Gaëlle Dosne^1^, Martin Bergstrand^1^, Mats O Karlsson^1^ |
| Author affiliations | ^1^Department of Pharmaceutical Biosciences, Uppsala University, P.O. Box 591, 751 24 Uppsala, Sweden |
| Corresponding author | Anne-Gaëlle Dosne: [annegaelle.dosne@farmbio.uu.se](mailto:annegaelle.dosne@farmbio.uu.se) |

Caption: Dashed vertical lines correspond to an additive error model (scedasticity=0) and a proportional error model (scedasticity=1). Colored shapes represent the approximated scedasticity on the untransformed scale with each of the three error model dTBS ($\boldsymbol{\lambda}$ and ζ), Box-Cox ($\boldsymbol{\lambda}$ only) and Power (ζ only). Horizontal full lines represent the span of scedasticity estimates across the three error models.


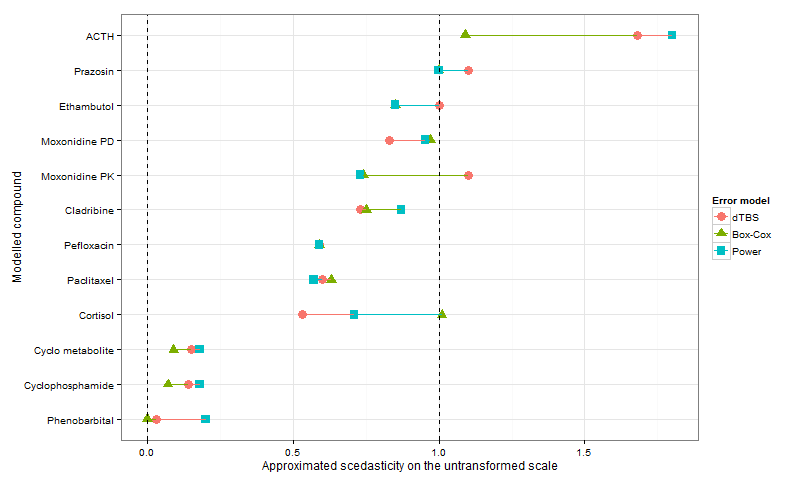

Supplement: Supplementary file 5 — Supplementary material 5 (DOCX 24 kb) [file 10928_2015_9460_MOESM5_ESM.docx]
