# Supplementary material for: A strategy for residual error modeling incorporating scedasticity of variance and distribution shape
Source: J Pharmacokinet Pharmacodyn. 2015 Dec 17;43:137–51. doi: 10.1007/s10928-015-9460-y (PMC4791481; doi:10.1007/s10928-015-9460-y)
Supplement: Supplementary file 6 — Supplementary material 6 (DOCX 238 kb) [file 10928_2015_9460_MOESM6_ESM.docx]

## **Online Resource 6:** **Influence of the different individuals on ΔOFV for the moxonidine PK and paclitaxel dTBS models**

| Article title | A Strategy for Residual Error Modeling Incorporating Scedasticity of Variance and Distribution Shape |
| --- | --- |
| Journal name | Journal of Pharmacokinetics and Pharmacodynamics |
| Author names | Anne-Gaëlle Dosne^1^, Martin Bergstrand^1^, Mats O Karlsson^1^ |
| Author affiliations | ^1^Department of Pharmaceutical Biosciences, Uppsala University, P.O. Box 591, 751 24 Uppsala, Sweden |
| Corresponding author | Anne-Gaëlle Dosne: [annegaelle.dosne@farmbio.uu.se](mailto:annegaelle.dosne@farmbio.uu.se) |

Caption: The grey dashed line represents the total ΔOFV between the original and the dTBS model. The red dashed line represents the significance level (degree of freedom of 2). Differences in individual OFV (OFV_i_) are computed, ranked in descending order (greatest absolute differences first) and subtracted from the total ΔOFV one by one along the x-axis according to their sign. Individuals who show improved fits with the dTBS approach are in blue, individuals who show deteriorated fits are in pink.

| 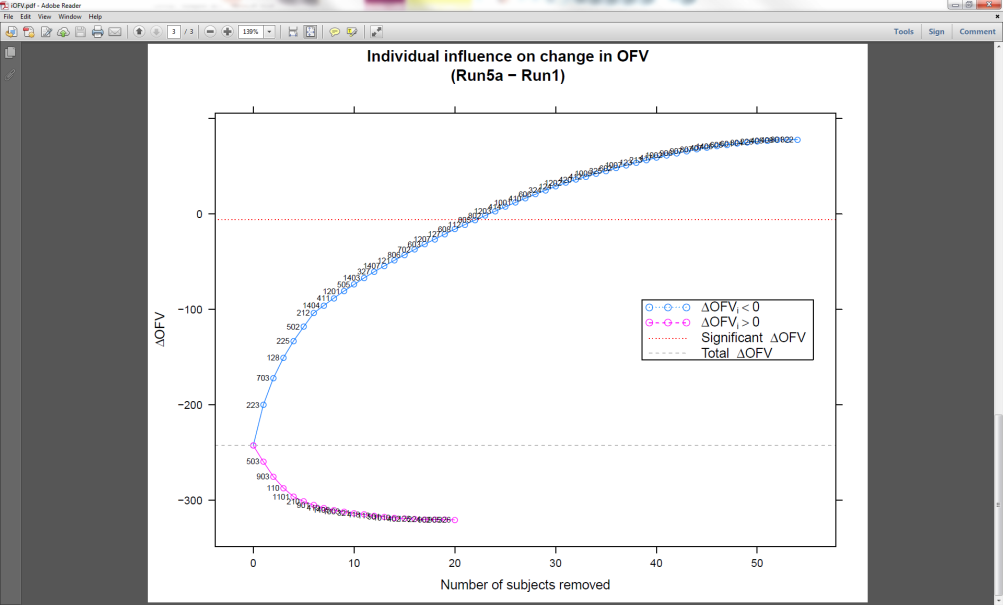(a) Moxonidine PK | (b) Paclitaxel PD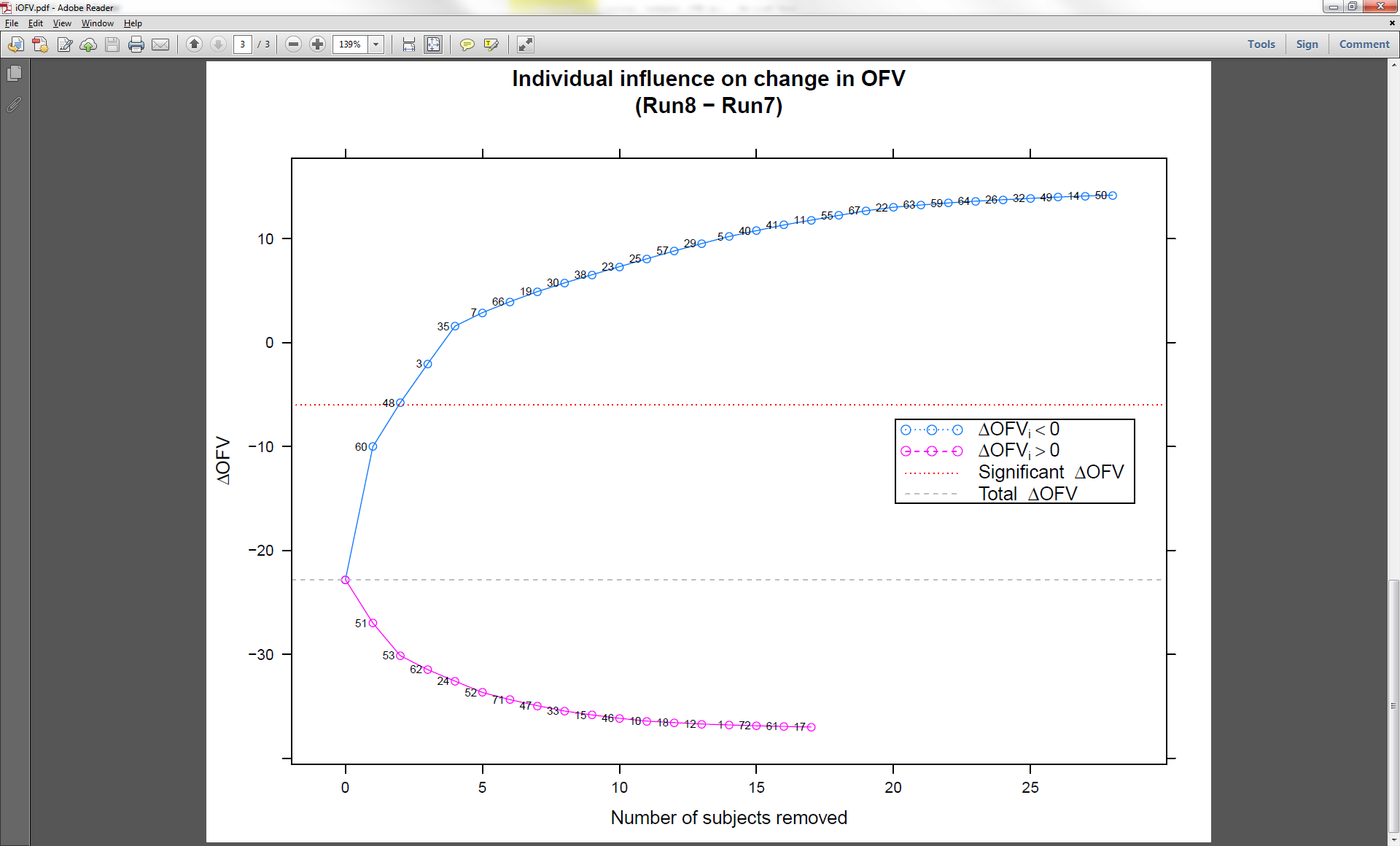 |
| --- | --- |
