# Supplementary material for: A strategy for residual error modeling incorporating scedasticity of variance and distribution shape
Source: J Pharmacokinet Pharmacodyn. 2015 Dec 17;43:137–51. doi: 10.1007/s10928-015-9460-y (PMC4791481; doi:10.1007/s10928-015-9460-y)
Supplement: Supplementary file 7 — Supplementary material 7 (DOCX 17 kb) [file 10928_2015_9460_MOESM7_ESM.docx]

## **Online Resource 7: Combined dTBS and t-distribution model file (prazosin example)**

| Article title | A Strategy for Residual Error Modeling Incorporating Scedasticity of Variance and Distribution Shape |
| --- | --- |
| Journal name | Journal of Pharmacokinetics and Pharmacodynamics |
| Author names | Anne-Gaëlle Dosne^1^, Martin Bergstrand^1^, Mats O Karlsson^1^ |
| Author affiliations | ^1^Department of Pharmaceutical Biosciences, Uppsala University, P.O. Box 591, 751 24 Uppsala, Sweden |
| Corresponding author | Anne-Gaëlle Dosne: [annegaelle.dosne@farmbio.uu.se](mailto:annegaelle.dosne@farmbio.uu.se) |

**Caption**: Example NONMEM model file using both dTBS and a t-distribution. Both methods are hard-coded within the model file. (As of now PsN does not produce this kind of models in an automated manner).

$PROBLEM prazosin data intraindividual variability

;; dTBS and Student distribution on RUV

;; DF = est

;; dTBS LAMBDA=&DELTA=est

$INPUT ID AGE RACE HT HCTZ AMT TIME DV RATE EVID SS II SID OCC

$DATA praz4.dta IGNORE=@

$SUBROUTINE ADVAN2 TRANS2

$PK

OCC2=1-OCC ;OCC

RR=0 ;RACE as binary covariate on CL

IF (RACE.EQ.2) RR=1

TMP=(HT-160)*THETA(1) ;HT as centered covariate on CL

IF (TMP.LE.0.) TMP=0.0

TMP2=(AGE-60)*THETA(2) ;AGE as centered covariate on CL

IF (AGE.LE.60.) TMP2=0.0

TVCL=THETA(3)+TMP-TMP2 ; TVCL dep on HT and AGE

TVCL=TVCL+THETA(4)*RR+THETA(5)*HCTZ ; TVCL dep on HT and AGE and RACE and HCTZ

IF (TVCL.LE.0.) EXIT 1

TVV=THETA(6)+HCTZ*THETA(7) ; TVV dep on HCTZ

IF (TVV.LE.0.) EXIT 1

TVKA=THETA(8)

CL=TVCL*EXP(ETA(3)*OCC+ETA(5)*OCC2+ETA(1)) ; IIV and IOV on CL

V =TVV *EXP(ETA(4)*OCC+ETA(6)*OCC2+ETA(2)) ; IIV and IOV on V

KA=TVKA*EXP(ETA(8)*OCC+ETA(9)*OCC2+ETA(7)) ; IIV and IOV on KA

S2=V

$ERROR

IPR1 = F

IF(F.LE.0) IPR1 = .0000001

;-------------- dTBS ------------------------------

LAMBDA = THETA(11) ; Box-Cox parameter

ZETA = LAMBDA + THETA(12) ; power

W = THETA(9)*IPR1**ZETA

IPRED = IPR1

IPRTR=IPRED

IF (LAMBDA .NE. 0 .AND. IPRED .NE.0) THEN

IPRTR=(IPRED**LAMBDA-1)/LAMBDA

ENDIF

IF (LAMBDA .EQ. 0 .AND. IPRED .NE.0) THEN

IPRTR=LOG(IPRED)

ENDIF

IF (LAMBDA .NE. 0 .AND. IPRED .EQ.0) THEN

IPRTR=-1/LAMBDA

ENDIF

IF (LAMBDA .EQ. 0 .AND. IPRED .EQ.0) THEN

IPRTR=-1000000000

ENDIF

IPRED=IPRTR

IRES = DV-IPRED

IWRES = IRES / W

IWRTR=IWRES

IF (LAMBDA.NE.0 .AND. DV.NE.0 .AND. W.NE.0) THEN

IWRTR=((DV**LAMBDA-1)/LAMBDA-IPRED)/W

ENDIF

IF (LAMBDA.EQ.0 .AND. DV.NE.0 .AND. W.NE.0) THEN

IWRTR=(LOG(DV)-IPRED)/W

ENDIF

IF (LAMBDA.NE.0 .AND. DV.EQ.0 .AND. W.NE.0) THEN

IWRTR=(-1/LAMBDA-IPRED)/W

ENDIF

IF (LAMBDA.EQ.0 .AND. DV.EQ.0 .AND. W.NE.0) THEN

IWRTR=(-1000000000-IPRED)/W

ENDIF

IWRES=IWRTR

;-------------- t-dist ------------------------------

DF = THETA(10) ; degrees of freedom of Student distribution

SIG1 = W ; scaling factor for standard deviation of RUV

PHI=(DF+1)/2 ; Nemes approximation of gamma funtion (2007) for first factor of t-distrib (gamma((DF+1)/2))

INN=PHI+1/(12*PHI-1/(10*PHI))

GAMMA=SQRT(2*3.14159265/PHI)*(INN/EXP(1))**PHI

PHI2=DF/2 ; Nemes approximation of gamma funtion (2007) for second factor of t-distrib (gamma(DF/2))

INN2=PHI2+1/(12*PHI2-1/(10*PHI2))

GAMMA2=SQRT(2*3.14159265/PHI2)*(INN2/EXP(1))**PHI2

COEFF=GAMMA/(GAMMA2*SQRT(DF*3.14159265))/SIG1 ; coefficient of PDF of t-distribution

BASE=1+IWRES*IWRES/DF ; base of PDF of t-distribution

IF(BASE.EQ.0) BASE=0.000001

POW=-(DF+1)/2 ; power of PDF of t-distribution

L=COEFF*BASE**POW ; PDF of t-distribution

Y = -2*LOG(L) - 2*(LAMBDA-1)*LOG(DV)

$THETA .0141 FIX ; 1. Coefficient on centered HT for CL

$THETA 0.601843 ; 2. Coefficient on centered AGE for CL

$THETA 21.8287 ; 3. TVCL without covariates

$THETA 1.03555 ; 4. Coefficient on RR for CL

$THETA -3.84579 ; 5. Coefficient on HCTZ for CL

$THETA 98.0459 ; 6. TVV without covariates

$THETA -24.6984 ; 7. Coefficient on HCTZ for V

$THETA 1.47414 ; 8. TVKA

$THETA 0.0953689 ; 9. RES ERROR

$THETA (3,5,200) ; 10. DF

$THETA (-5,1.22821,5) ; 11. LAMBDA

$THETA (-5,-0.00773397,5) ; 12. DELTA

$OMEGA BLOCK(2)

0.0868132 ; 1. IIV CL

0.0880643 0.0997573 ; 2. IIV V

$OMEGA BLOCK(2)

0.0350799 ; 3. IOV CL OCC1

0.0312407 0.0292565 ; 4. IOV V OCC1

$OMEGA BLOCK(2) SAME; 6. IOV V OCC2

$OMEGA 0.270856 ; 7. IIV KA

$OMEGA BLOCK(1)

0.445329 ; 8. IOV KA OCC1

$OMEGA BLOCK(1) SAME

$ESTIMATION NOABORT MAXEVAL=9999 -2LL METH=1 LAPLACE PRINT=10 ;MSFO=msf

$TABLE ID TIME EVID IPRED IWRES CWRES OCC NOPRINT FILE=sdtab6
